# Supplementary material for: Self assembling cluster crystals from DNA based dendritic nanostructures
Source: Nat Commun. 2021 Dec 9;12:7167. doi: 10.1038/s41467-021-27412-3 (PMC8660878; doi:10.1038/s41467-021-27412-3)
Supplement: Supplementary file 1 — Supplementary Information [file 41467_2021_27412_MOESM1_ESM.pdf]

Supplementary Information

Self assembling cluster crystals from DNA based dendritic nanostructures

Emmanuel Stiakakis[1, *], Niklas Jung[2], Nataša Adžić[3], Taras Balandin[4], Emmanuel Kentzinger[5], Ulrich Rücker[5], Ralf Biehl[6], Jan K. G. Dhont[1,6], Ulrich Jonas[2], and Christos N. Likos[3, *]

[1]Biomacromolecular Systems and Processes, Institute of Biological Information Processing (IBI-4), Forschungszentrum Jülich, D-52425 Jülich, Germany

[2]Macromolecular Chemistry, Department Chemistry-Biology, University of Siegen, D-57076 Siegen, Germany

[3]Faculty of Physics, University of Vienna, Boltzmanngasse 5, A-1090 Vienna, Austria

[4]Structural Biochemistry, Institute of Biological Information Processing (IBI-7), Forschungszentrum Jülich, D-52425 Jülich, Germany

[5]Jülich Centre for Neutron Science JCNS and Peter Grünberg Institut PGI, JARA-FIT, Forschungszentrum Jülich, D-52425 Jülich, Germany

[6]Neutron Scattering and Biological Matter (JCNS-1/IBI-8), Forschungszentrum Jülich, D-52425 Jülich, Germany

[7]Heinrich-Heine-Universität Düsseldorf, Universitätsstraße 1, D-40225 Düsseldorf, Germany

[*]Corresponding authors: e.stiakakis@fz-juelich.de; christos.likos@univie.ac.at

This PDF file includes:

Supplementary Figs. 1 to 5
Supplementary Notes 1 to 4
Supplementary Methods 1 to 3
Supplementary References 1 to 6

Supplementary Figures

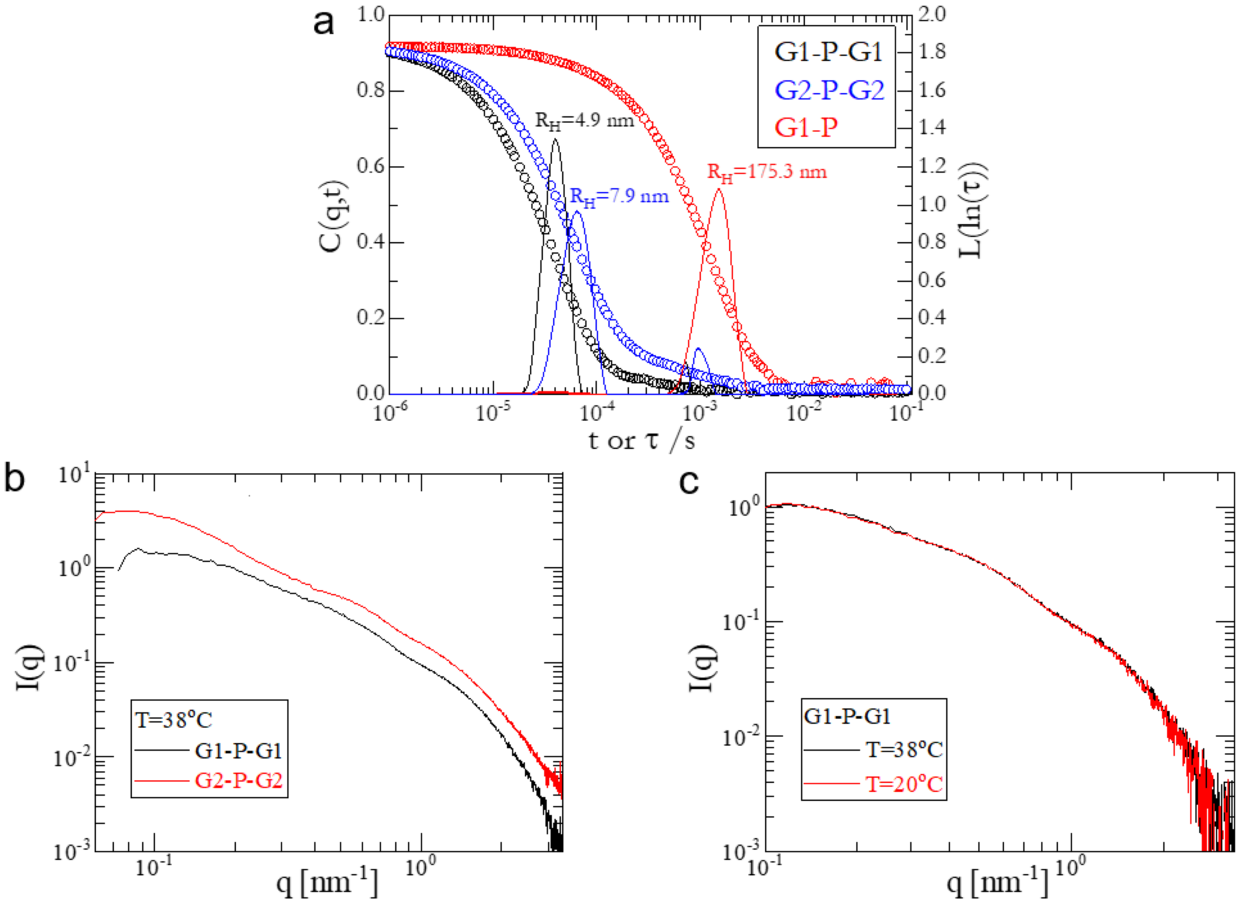

Supplementary Figure 1. a, Intermediate scattering functions $C(q,t)$ at a scattering angle of $\theta = 90^{\circ}$ ($q = 0.001875\ \text{nm}^{-1}$) of dilute G1-P-G1, G2-P-G2 and G1-P aqueous solutions ($c_{\text{salt}} = 150\text{mM NaCl}$) solutions at a concentration $c = 5.0\ \text{mg/ml}$ and $T = 38^{\circ}\text{C}$. The corresponding distribution functions $L(\ln\tau)$ are also shown (solid-lines), together with the extracted hydrodynamic radius R_{H}. **b,** The SAXS form factors of G1-P-G1 and G2-P-G2 at $T = 38^{\circ}\text{C}$. **c,** 1D-SAXS profiles of dilute G1-P-G1 solution ($c = 2.5\ \text{mg/ml}$, $c_{\text{salt}} = 150\text{mM NaCl}$) at temperatures below (20°C) and above (38°C) the Poxa's LCST.

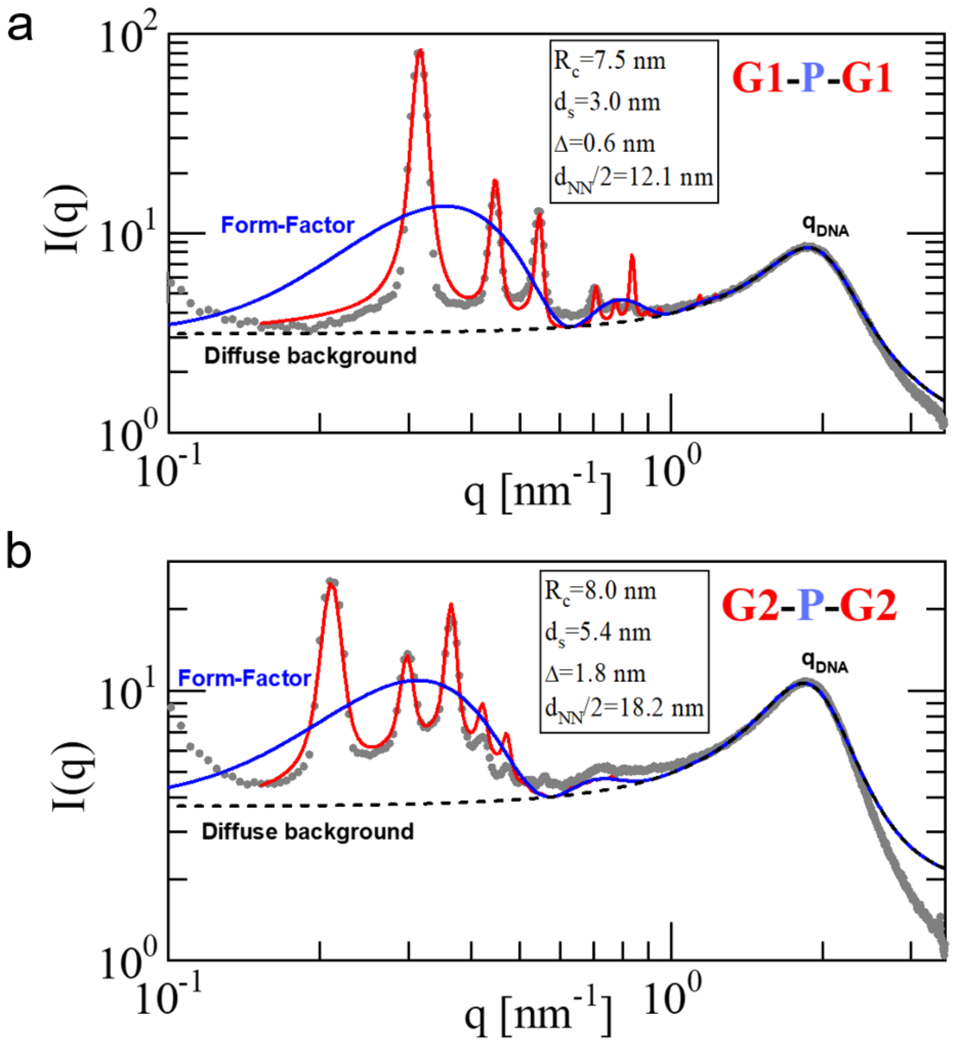

Supplementary Figure 2. Fitting of the 1D-SAXS profiles (gray dots) for **(a)** G1-P-G1 ($c = 255.7$ mg/ml and $T = 38°$C) and **(b)** G2-P-G2 ($c = 220.5$ mg/ml and $T = 33°$C). The model fits (red lines) have contributions from a BCC lattice of core-shell particles (blue lines corresponding form-factors including background) and a background due to the inhomogeneous distribution of DNA fragments. The difference between core-shell particle size and next neighbor distance d_{NN} allows position fluctuations with a root mean square width $\sqrt{\langle r^2 \rangle} \equiv \Delta$ for G2-P-G2 while these are strongly suppressed for G1-P-G1. Correspondingly, G1-P-G1 shows more high order peaks which are suppressed for G2-P-G2 due to the Debye-Waller factor. For G1-P-G1 the (022) peak (fourth reflection) is hidden because of the minimum in the form-factor. More details about the fitting parameters and procedures are presented in Supplementary Note 2.

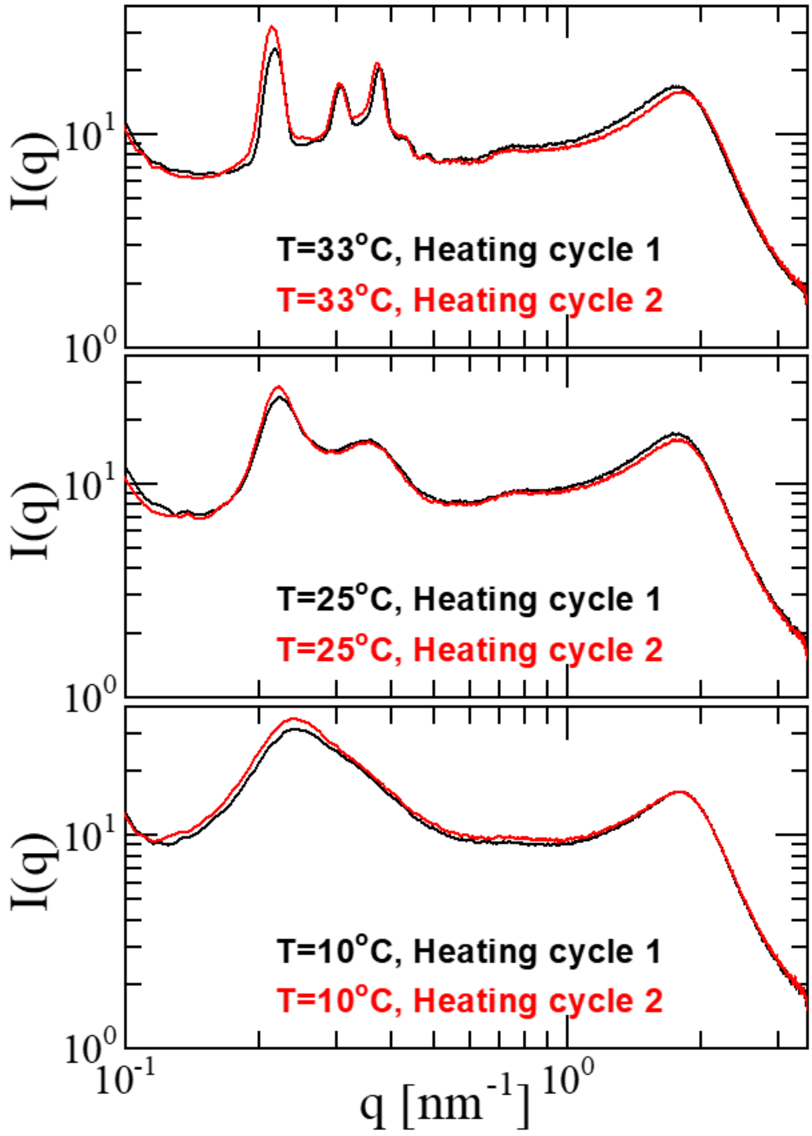

Supplementary Figure 3. Temperature-dependent 1D-SAXS profiles of the G2-P-G2 at DNA concentration of $c = 204.73$ mg/ml. Bottom panel: Cluster Fluid, Middle panel: Onset of order, Top panel: BCC Cluster. The colors indicate the thermal history of the sample. Each scattering profile was acquired after annealing for at least 10 min at each temperature to ensure thermal equilibrium.

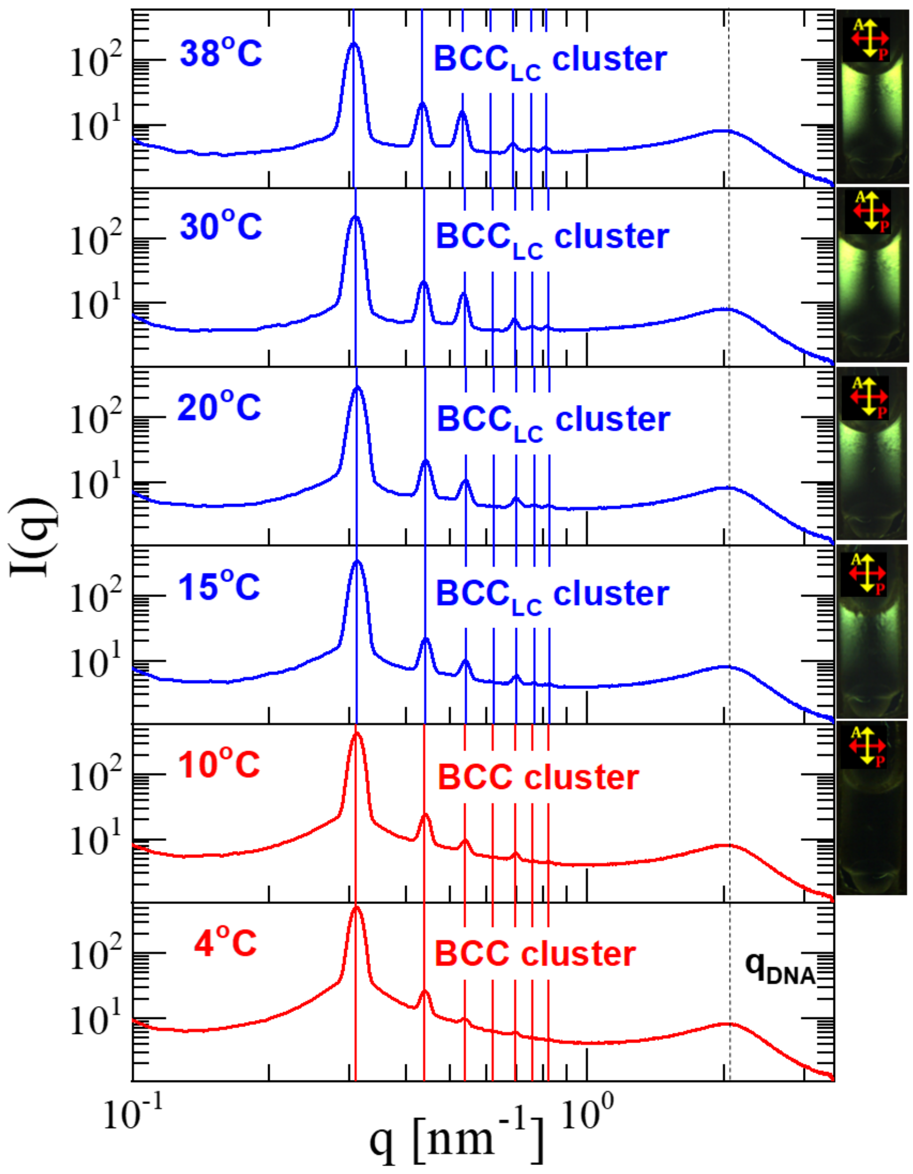

Supplementary Figure 4. Temperature-dependent SAXS profiles of the G1-P-G1 at $c = 300.8$ mg/ml. The corresponding depolarized images of the sample are also depicted. The vertical solid-lines indicate the positions of the first seven allowed reflections for a BCC crystal. The black dashed-line is guide for the temperature-dependence of the q_{DNA} peak.

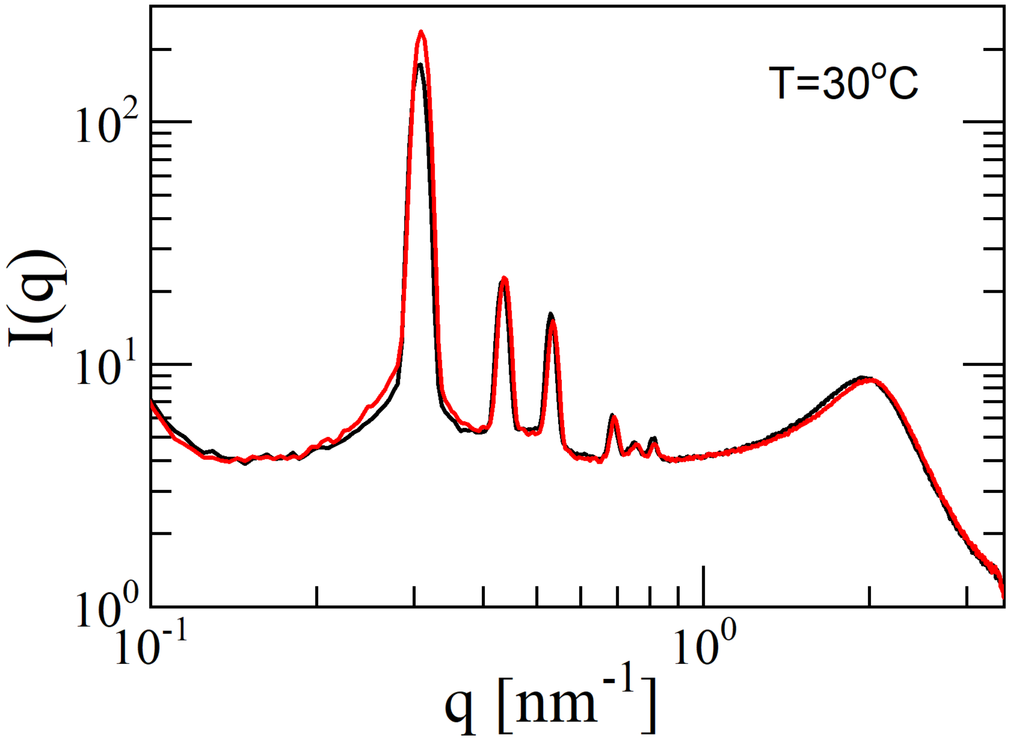

Supplementary Figure 5. 1D-SAXS profiles of the G1-P-G1 at concentration of $c =$ 300.8 mg/ml for $T = 30°$C. The red profile was acquired six months after the black profile.

Supplementary Notes

Contents

1. **Self-assembly properties of G1-P-G1, G2-P-G2 and G1-P in dilute solutions**
2. **General fitting procedure of 1D-SAXS profiles**
   2.1 Fitting parameters
   2.2 SAXS modeling
   2.3 A note on modeled G1-P-G1 and G2-P-G2 cluster form-factors
3. **Temperature-induced cluster Fluid-to-BCC transition in G1-P-G1**
4. **On the origin of liquid crystalline BCC-like (BCC_{LC}) in G1-P-G1 system**

Supplementary Note 1

Self-assembly properties of G1-P-G1, G2-P-G2 and G1-P in dilute solutions

Light scattering (LS) and Small-Angle X-ray scattering (SAXS) measurements were performed on dilute aqueous salt solutions (150 mM NaCl) of G1-P-G1, G2-P-G2 and G1-P at temperatures below and well-above the LCST of Poxa. The translation diffusion coefficient D_0 obtained from the normalized intermediate scattering function $C(q,t) = e^{-D_0 q^2 t}$, with q the scattering vector, yields the hydrodynamic radius $R_H = k_B T/(6\pi\eta_0 D_0)$, where η_0 is the solvent viscosity and k_B is Boltzmann's constant. For temperatures well-above the LCST of the Poxa-block, we obtain $R_H = 4.9\,\text{nm}$ for G1-P-G1, $R_H = 7.9\,\text{nm}$ for G2-P-G2 and $R_H = 175.3\,\text{nm}$ for G1-P-G1 (Supplementary Fig.1a). In full agreement with the LS data, SAXS measurements on dilute G1-P-G1 and G2-PG2 aqueous solutions reveal that the global size and internal structure of our DNA-based triblocks is temperature-insensitive, with the radii of gyration being $R_g = 6.4$ nm for G1-P-G1 and $R_g = 9.8$ nm for G2-P-G2. The R_g values were obtained from the Guinier plot analysis of the SAXS profiles presented in Supplementary Fig. 1b-c.

Supplementary Note 2

General fitting procedure of 1D-SAXS profiles

2.1 Fitting parameters

Parameters resulting from SAXS data fit for the G1-P-G1 and G2-P-G2 presented in Supplementary Fig. 2a and Supplementary Fig. 2b, respectively.

	**G1-P-G1**	**G2-P-G2**
domain size D	344.0±12.0 nm	333.0±11.0 nm
lattice constant, a_{BCC}	27.9±0.2 nm	42.0±0.2 nm
root mean square displacement, Δ	0.6±0.4 nm	1.8±0.6 nm
Half of the next neighbor distance along the room diagonal, $d_{NN}/2$	12.1 nm	18.2nm
ratio scattering length density shell/core	-0.5±0.4	-0.28±0.1
core radius, R_c	7.5±0.7 nm	8.0±0.3 nm
shell radius, d_s	3.0±1.8 nm	5.4±1.0 nm
Correlation distance d	1.9±0.1 nm	2.5±0.1 nm
Correlation length ξ	2.1±0.6 nm	1.9±0.1 nm

2.2 SAXS modeling

The SAXS scattering patterns of DNA clusters at high concentrations show a prominent peak structure that corresponds to a BCC lattice with a pronounced unstructured and broad peak at higher scattering vectors q. The overall pattern is described by the scattering of a crystal domain $I_c(q)$ with a nonconstant background $I_b(q)$ as $I(q)=I_c(q)+I_b(q)$.

The scattering intensity of a crystal domain in powder average is

$$I_c(q) = nP(q)S(q)$$

with particle density n.

The structure factor $S(q)$ is expresses as [1]:

$$S(q) = 1 + (Z_0(q) - 1)DW(q)$$

$$Z_0(q) = \frac{(2\pi)^2 c}{n_u v_d q^2} \sum_{hkl} m_{hkl} f_{hkl}^2 L_{hkl}(q)$$

with Miller indices hkl numbering the Bragg peaks, n_u being the number of particles in the unit cell, f^2_{hkl} as unit cell structure factor considering extinction rules, v_d as volume of the unit cell, Porod constant $c \approx 1$ and peak multiplicity m_{hkl}.

The Debye-Waller factor $DW(q) = e^{-q^2\langle r^2\rangle}$ depends on thermal dislocations with mean square fluctuations $\langle r^2\rangle = \Delta$ and reduces peak intensities for increasing q.

As peak shape function $L_{hkl}(q)$ we use a Gauss function. According to the Debye-Scherrer equation, the width of the Gaussian $fwhm = \frac{2\pi}{D}$ is related to the domain size D [2].

For a BCC lattice, the next neighbor distance d_{NN} along the room diagonal can be calculated from the BCC lattice constant a_{BCC} as $d_{NN} = \left(\frac{\sqrt{3}}{2}\right) a_{BCC}$ which for a close packed BCC lattice corresponds to the corresponding maximum sphere radius.

The form factor of DNA clusters is modeled as core-shell particle with polymer linkers in the core and a shell filled by DNA fragments, a schematic of which is shown in the right cartoon of Fig. 3f. The core-shell form factor is $P(q) = \left(\sum_n f_{a,n}\left(q, R_{i,n}, R_{o,n}\right)\right)^2$ with the sum running over n = *core, shell*, and the scattering amplitude $f_{a,n}$ of shell, respectively core (with R_i=0) as [3] :

$$f_{a,n}\left(q, R_{i,n}, R_{o,n}\right) = \frac{4\pi}{3} \Delta\rho_n \, r^3 \frac{3\left(\sin(qr) - qr\cos(qr)\right)}{(qr)^3}\Bigg|_{r=R_{i,n}}^{r=R_{o,n}}$$

Here R_i, R_o are the inner and outer radius and $\Delta\rho_n$ is the scattering contrast to the solvent of shell, respectively core.

The low intensity at low q suggests that the scattering contrast $\Delta\rho$ between core and shell has opposite sign to result in a condition close to matching of the core against the shell at low q with $V_{shell}\Delta\rho_{shell} \approx -V_{core}\Delta\rho_{core}$.

The broad peak around 2 nm^{-1} is attributed to correlations between DNA fragments that are not covered in the BCC core-shell model. This background is described by the Teubner-Strey model [4]:

$$I_b(q) = \frac{8\pi\eta^2/\xi}{a_2 + 2bq^2 + q^4}$$

$$k = \frac{2\pi}{d}; a_2 = (k^2 + \xi^{-2})^2; b = k^2 - \xi^{-2}$$

That describes the scattering of density correlations like $\gamma(r) = \frac{d}{2\pi r} e^{-\frac{r}{\xi}} \sin\left(\frac{2\pi r}{d}\right)$ with correlation length ξ and characteristic periodicity d. η is the characteristic contrast variation. We assume that the peak maximum correlates to DNA fragment distances with $q_{max}^2 = \left(\frac{2\pi}{d}\right)^2 - \xi^{-2}$.

2.3 A note on modeled G1-P-G1 and G2-P-G2 cluster form-factors

We would like to attract the attention to a particularly interesting observation concerning the shape of the form-factors in the q-region at which the higher-order reflections are observed. For the G1-P-G1, the first minimum of the cluster form-factor is located exactly at the q-position of the fourth-order Bragg peak (blue curve in Supplementary Fig. 2a) and, therefore explains the absence of this reflection in our 1D-SAXS profiles (second panel and third panel from the bottom of Fig. 4a and Fig. 4b, respectively). It is also worth mentioning that the lack of the above-mentioned reflection allowed us to carefully adjust our fitting parameters. In addition, and contrary to the G1-P-G1 system, the second maximum of the G2-P-G2 cluster form-factor (blue curve in Supplementary Fig. 2b) is not superimposed onto the higher order Bragg peaks arising from the BCC lattice, and, hence, its presence and location can clearly be discerned in the experimental data. Interestingly, the position of the G2-P-G2 cluster form-factor peak is not markedly affected by the DNA concentration or temperature, indicating that the cluster size remains invariant under such changes.

Supplementary Note 3

Temperature-induced cluster Fluid-to-BCC transition in G1-P-G1.

In Fig. 4b, a cluster fluid-to-crystal transition is presented for the G1-P-G1 at DNA concentration $c = 255.7$ mg/ml, through alteration of temperature. Up to 20°C, a temperature well below the LCST of the Poxa chain, the G1-P-G1 solution exhibits a cluster fluid structure which progressively becomes more ordered by increasing the temperature. A slight further temperature increase induces a markedly different phase, as can be seen from the SAXS profile acquired at 23°C. Comparison of the observed Bragg peaks with the first seven allowed reflections for a BCC lattice with $\alpha_{BCC} = 28.2$ nm (red vertical lines) offers a compelling evidence regarding the morphology of this cluster crystal structure. This fluid-to-crystal transition is in accordance with the self-assembly of our dendritic based triblocks into clusters with a temperature-dependent aggregation number. Since the segregation strength between the Poxa and the DNA in our dendritic-based triblocks becomes stronger with increasing temperature, the observations are compatible with an overall cluster size growth through a simultaneous swelling of its core (Poxa chains stretching) and an increase of the aggregation number. This explains why no significant change is seen in neither the q_{DNA}-peak position nor in its width over the temperature range between 4°C and 23°C.

Supplementary Note 4

On the origin of liquid crystalline BCC-like (BCC_{LC}) in G1-P-G1 system.

The two bottom panels of Fig. 7a and the depolarized images of the samples presented in the top panel of Fig. 7b indicate that a transition from BCC-to-BCC_{LC} occurs over a DNA concentration range from 287.7 mg/ml to 300.8 mg/ml, without, however, a change in the BCC lattice constant. Further augmentation of G1-P-G1 concentration leads to a gradual increase in lattice constant by approximately 8% going from 300.8 mg/ml to 337.7 mg/ml (top panel of Fig. 7b). The sample exhibits a strong and colourful birefringence under crossed-polarizers (rightmost image in the top panel of Fig. 7b). In addition, the SAXS profiles of the most concentrated birefringent BCC-like samples show the so-far missing fourth Bragg reflection (marked by the grey circles in the middle panel of Fig. 7a), suggesting that the BCC-to-BCC_{LC} transition is followed by a change in shape or size of the clusters. On the basis of the above results, and in conjunction with the requirement of releasing the packing frustration as the almost close packed BCC crystal is further compressed, we surmise that the optical anisotropy of the BCC_{LC} phase can be ascribed to orientationally ordered ellipsoidal-like clusters occupying the sites of the BCC lattice (Fig. 7c). Such a molecular packing scenario is plausible since a nematic-like "cubic" phase may exhibit optical birefringence without a departure from cubic symmetry. Furthermore, we expect that for a small energy cost, a cluster deformation can be allowed, stemming from its moderate occupancy character. In the BCC_{LC} cluster crystal regime (blue region in the phase diagram of Fig. 6a), an increase of N_{occ} from 38 to 53 is found by increasing the G1-P-G1 concentration (bottom panel of Fig. 7b). It is imperative to note that efforts to refine the unit-cell parameters have shown that the changes were too small to decisively demonstrate a non-cubic symmetry, such as in a tetragonal lattice. However, the G1-P-G1 packing scenario remains a matter of speculation and further work is required for elaborating this conjecture.

Supplementary Methods

Contents

1. **DNA sequences**
2. **Synthesis procedure of poly(2-oxazoline)s**
   2.1 Synthesis of mono-azide Poxa (CH_3-P[(2-*n*-propyl-2-oxazoline)-*co*-(2-ethyl-2-oxazoline)]-N_3)
   2.2 Synthesis of di-azide Poxa (N_3-P[(2-*n*-propyl-2-oxazoline)-*co*-(2-ethyl-2-oxazoline)]-N_3)
3. **Supplementary spectra of poly(2-oxazoline)s**

Supplementary Method 1

DNA sequences

The DNA sequences and construction scheme for the all-DNA dendrons employed for the construction of the G1-P, G1-P-G1 and G2-P-G2 are given below from 5' to 3' end. The DBCO indicates the site-specific terminal oligo modification with a dibenzylcyclooctyne (DBCO) functional group. A TEG linker (Biomers) is introduced between the DBCO group and the oligo in order to reduce steric hindrance between azide-terminated polymer (Poxa) and DBCO-functionalized three-arm DNA (G1) junction. The bold letters correspond to sticky-end sequence, p indicates the position of the phosphate modification and T_5 stands for a poly(thymine) sequence with length of five (5) bases.

<u>DNA strands sequences:</u>

Y_{1a} : 5'-DBCO-TEG-(T_5) TGG ATC CGC ATG ACA TTC GCC GTA AG-3'

Y_{1b} : 5'-(T_5) CTT ACG GCG AAT GAC CGA ATC AGC CT-3'

Y_{1c} : 5'-(T_5) AGG CTG ATT CGG TTC ATG CGG ATC CA-3'

Y_{1b}^{P} : 5'-p-**TGAC** CTT ACG GCG AAT GAC CGA ATC AGC CT-3'

Y_{1c}^{P} : 5'-p-**TGAC** AGG CTG ATT CGG TTC ATG CGG ATC CA -3'

Y_{2a}^{P} : 5'-p-**GTCA** TGG ATC CGC ATG ACA TTC GCC GTA AG -3'

The theoretical molecular mass (M_w^{theor}) of each individual DNA strand and the corresponding experimental value (M_w^{exper}) as determined by Maldi-TOF mass spectroscopy are listed below. These values were provided by the supplier (Biomers).

Y_{1a} : M_w^{theor} = 10063 g/mol, M_w^{exper} = 10081 g/mol

Y_{1b} : M_w^{theor} = 9461 g/mol, M_w^{exper} = 9465 g/mol

Y_{1c} : M_w^{theor} = 9523 g/mol, M_w^{exper} = 9529 g/mol

Y_{1b}^P : M_w^{theor} = 9256 g/mol, M_w^{exper} = 9264 g/mol

Y_{1c}^P : M_w^{theor} = 9318 g/mol, M_w^{exper} = 9322 g/mol

Y_{2a}^P : M_w^{theor} = 9287 g/mol, M_w^{exper} = 9293 g/mol

G1-P and G1-P-G1 construction scheme:

1st generation: G1: Y_{1a}+ Y_{1b}+Y_{1c}

G2-P-G2 construction scheme:

1st generation: G1: Y_{1a}+ Y_{1b}^P + Y_{1c}^P

2nd generation: G2: G1 + 3Y_2 where Y_2=Y_{2a}^P+ Y_{1b}+ Y_{1c}

Supplementary Method 2

Synthesis procedure of poly(2-oxazoline)s

Method 2.1: Synthesis of mono-azide Poxa (CH_3-P[(2-*n*-propyl-2-oxazoline)-*co*-(2-ethyl-2-oxazoline)]-N_3)

The following synthetic scheme is employed according to Osawa et al. [5]:

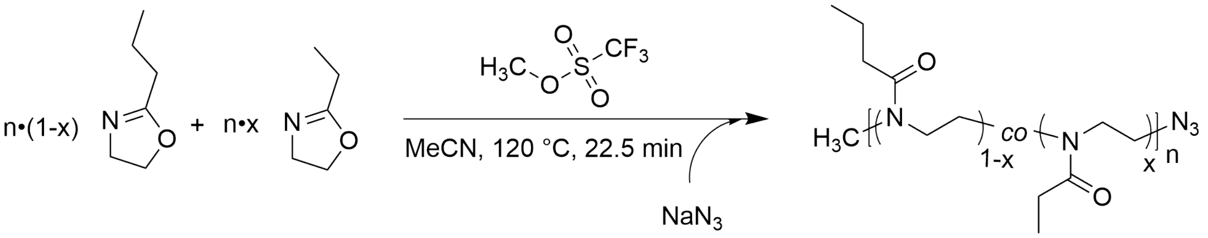

2-n-propyl-2-oxazoline (4509 mg, 39.85 mmol) and 2-ethyl-2-oxazoline (1538 mg, 15.51 mmol) were filled into a glass vial. To this solution methyl triflate (58.38 mg, 0.90 mmol), dissolved in anhydrous acetonitrile (15379 mg), was added. The polymerization was carried out in a microwave (T= 120°C ramp 2:00 min) for 22.5 min. Afterwards the mixture was cooled to room temperature and sodium azide (604 mg, 9.29 mmol) was added under argon atmosphere. The polymer solution was stirred at 70°C overnight. Afterwards, the solvent was removed and the residue re-dissolved in $CHCl_3$. The organic phase was washed with saturated aqueous $NaHCO_3$-solution (2x20 ml). Afterwards, the solution was concentrated and precipitated into ice-cold diethyl ether and dried in vacuum overnight. - Yield: 4020 mg (66%) - ^{1}H NMR (500

MHz, $CDCl_3$) δ 3.64 – 3.18 (601H), 3.06 – 2.98 (3H), 2.45 – 2.10 (301H), 1.81 – 1.72 (2H), 1.72 – 1.51 (215H), 1.16 – 1.00 (125H), 0.99 – 0.84 (322H). - IR (ATR): $\bar{\nu}$ = 3495, 2096, 1633, 1418, 1194 cm^{-1} ppm.

Analysis: ^1H NMR, 2-n-propyl-oxazoline:2-ethyl-2-oxazoline (72:28); GPC, RI: $\bar{M}_n$: 18.2 kg·mol^{-1}, $\bar{M}_w$: 19.4 kg·mol^{-1}, Đ: 1.07, M_p: 19.4 kg·mol^{-1}; UV/Vis, Cloud Point: (5.0 mg·ml^{-1}), LCST: 35.3 °C in salt-free aqueous solutions.

Method 2.2: Synthesis of di-azide Poxa (N_3-P[(2-*n*-propyl-2-oxazoline)-*co*-(2-ethyl-2-oxazoline)]-N_3)

The following synthetic scheme is employed:

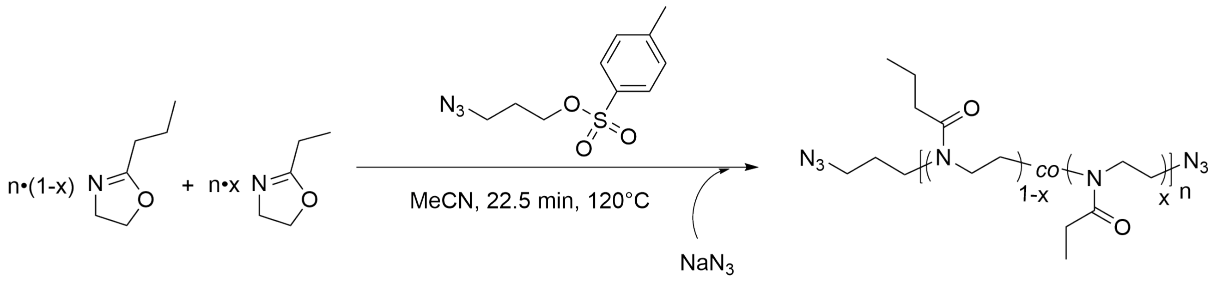

N_3-Prop-Tos, used as initiator for synthesis of N_3-P[(nPrOxa)-*co*-(EtOxa)]-N_3, was prepared according literature [6]. The polymerization was carried out under same conditions as described previously (see Supplementary Method 2.1). Instead of methyl triflate, azidopropyl tosylate (54.1 mg, 211 µmol) was used. Other masses: 2-n-propyl-2-oxazoline (2323 mg, 20.5 mmol), 2-ethyl-2-oxazoline (744.8 mg, 7.5 mmol) and anhydrous acetonitrile (8983 mg) -Yield: 1700 mg (54%)

Analysis: ^1H NMR, 2-n-propyl- oxazoline:2-ethyl-2-oxazoline (73:27); GPC, RI: $\bar{M}_n$: 13.7 kg·mol^{-1}, $\bar{M}_w$: 15.9 kg·mol^{-1}, Đ: 1.16, M_p: 16.7 kg·mol^{-1}; UV/Vis, Cloud Point: (5.0 mg·ml^{-1}), LCST: 35.1 °C in salt-free aqueous solutions.

Supplementary Method 3

Supplementary spectra of poly(2-oxazoline)s

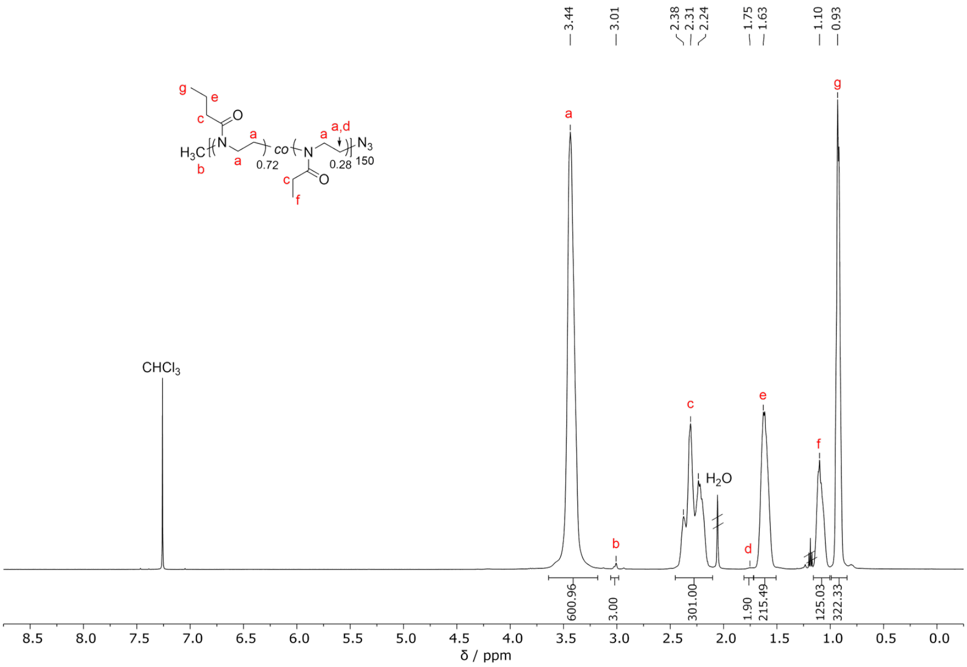

^1H NMR (500MHz) spectrum of mono-azide Poxa (CH_3-P[(2-n-propyl-2-oxazoline)-co-(2-ethyl-2-oxazoline)]-N_3), recorded in $CDCl_3$.

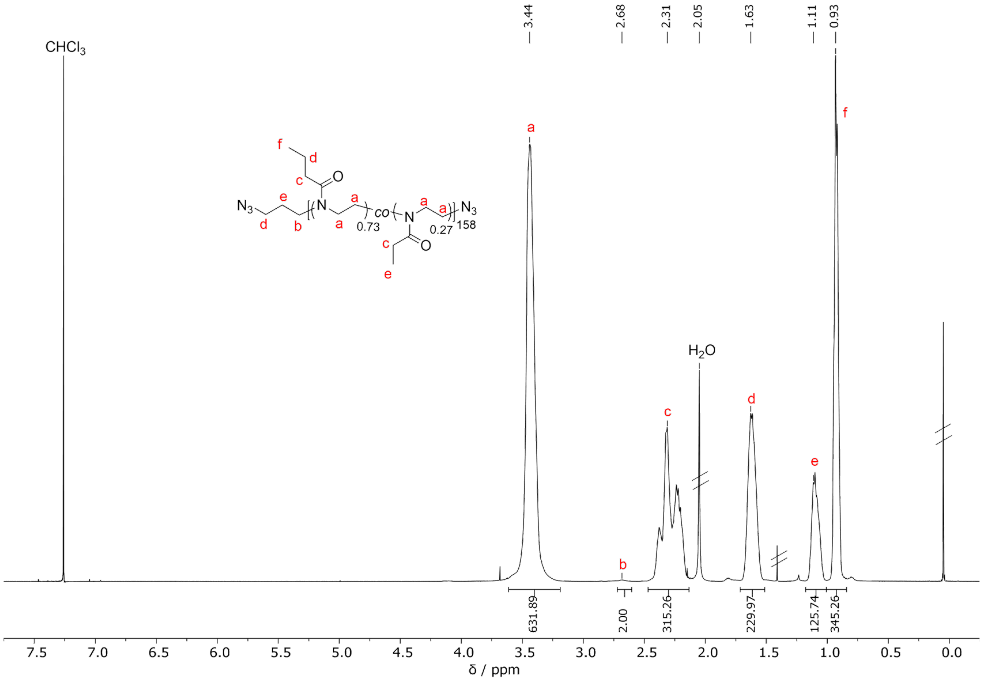

^1H NMR (500MHz) spectrum of di-azide Poxa (N_3-P[(2-n-propyl-2-oxazoline)-co-(2-ethyl-2-oxazoline)]-N_3), recorded in $CDCl_3$.

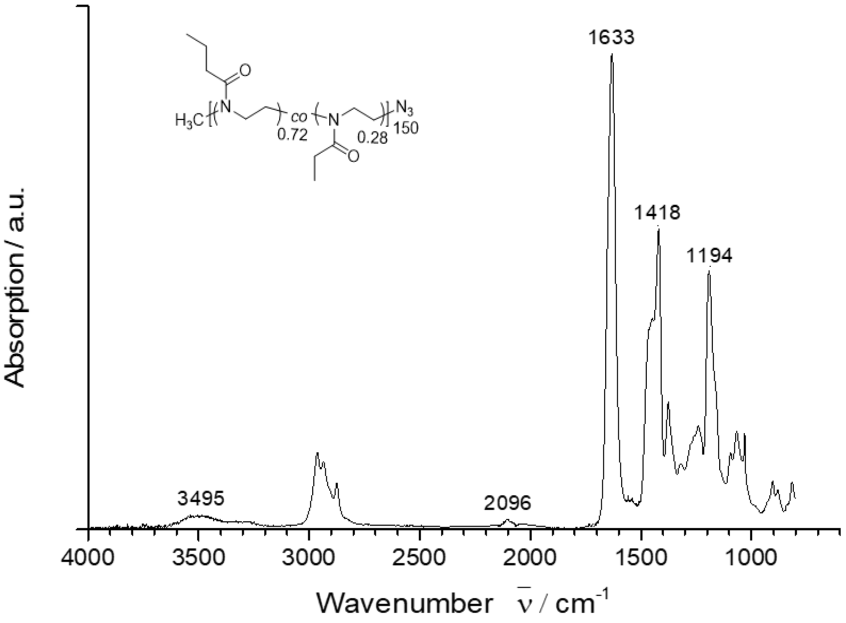

IR spectrum (ATR) of mono-azide Poxa (CH_3-P[(2-n-propyl-2-oxazoline)-co-(2-ethyl-2-oxazoline)]-N_3).

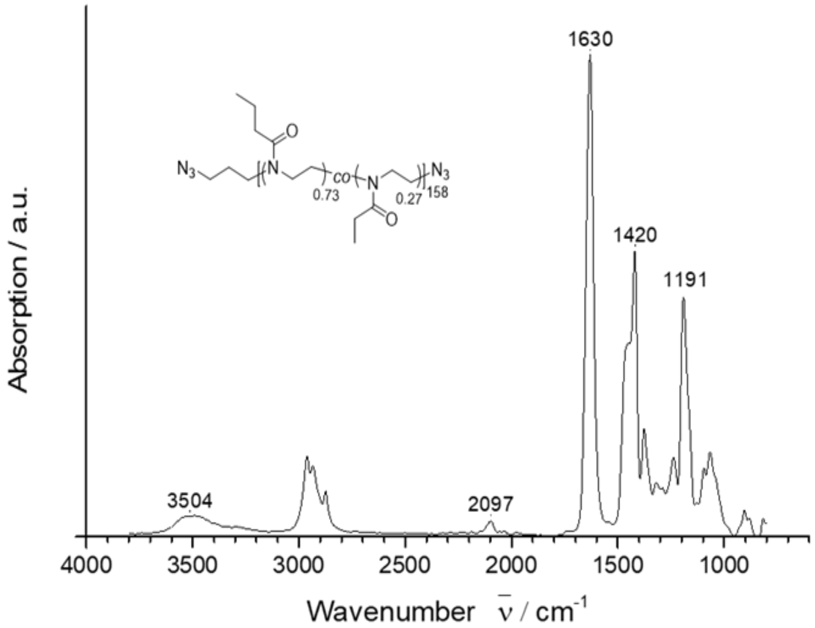

IR spectrum (ATR) of di-azide Poxa (N_3-P[(2-n-propyl-2-oxazoline)-co-(2-ethyl-2-oxazoline)]-N_3).

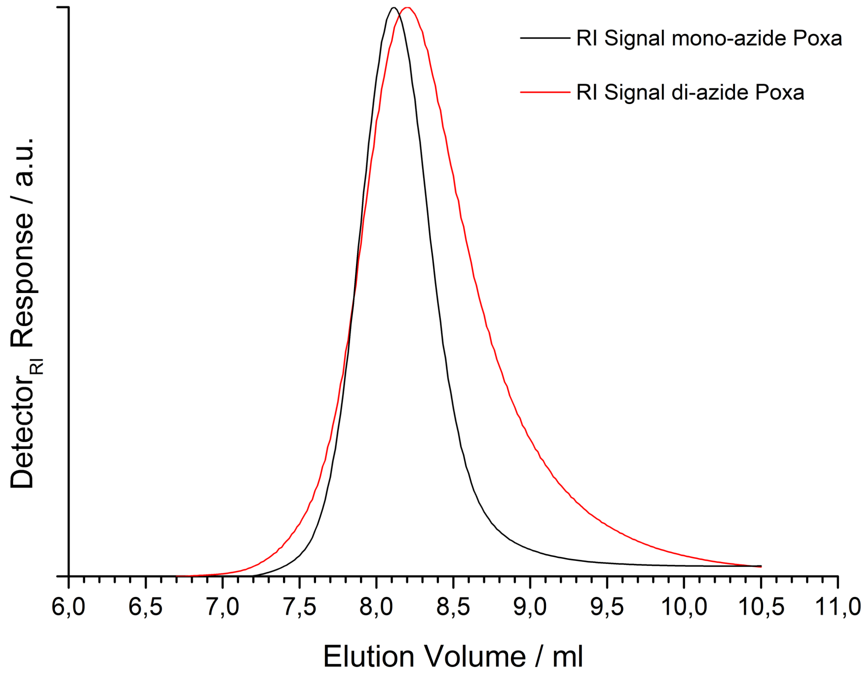

Normalized GPC Elugrams of mono-azide Poxa (black) and di-azide Poxa (red). Measuring conditions: PSS system (Agilent 1260) equipped with a Gram 100 Å column and a 10 µm particle size precolumn was utilized at $T = 60$ °C. As eluent dimethylacetamide (DMAc) mixed with $\beta = 1$ g·l^{-1} LiBr was used. 20 µl of the polymer-samples were injected. The flow rate of the system was 1 ml·min^{-1}. The calibration curve was measured using PMMA standards (PSS, Mainz).

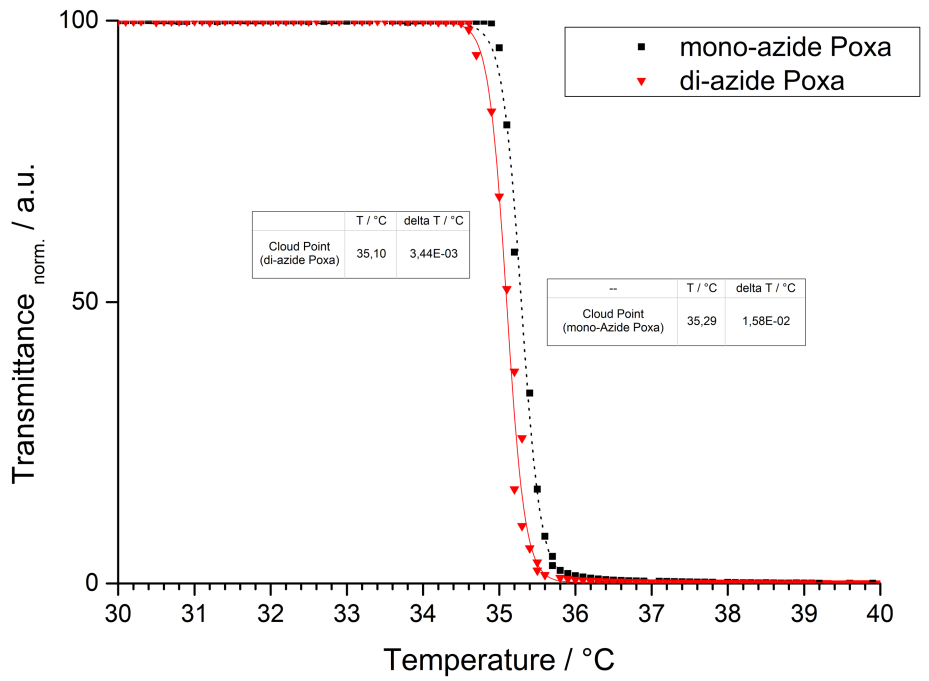

Normalized transmittance (λ = 600 nm) vs. temperature diagrams of mono-azide Poxa (black) and di-azide Poxa (red), measured at β = 5.0 mg/ml in salt free water (heating rate: 1 °C min^{-1}). The cloud point and the corresponding uncertainty ΔT were observed by fitting the data using nonlinear curve Boltzmann fit and determining the temperature at the inflection point of the heating curve.

Supplementary References

1. Förster, S., Timmann, A., Konrad, M., Schellbach, C., Meyer, A., Funari, S. S., Mulvaney, P. & Knott, R. Scattering Curves of Ordered Mesoscopic Materials. *J. Phys. Chem. B* **109**, 1347–1360 (2005).
2. Patterson, A. L. The Scherrer Formula for X-Ray Particle Size Determination. *Phys. Rev.* **56**, 978–982 (1939).
3. Guinier, A. & Fournet, G. Small-angle scattering of X-Rays. (John Wiley and Sons, New York, 1995).
4. Teubner, M. & Strey, R. Origin of the Scattering Peak in Microemulsions. *J. Chem. Phys.* **87**, 3195–3200 (1987).
5. Osawa, S., Ishii, T., Takemoto, H., Osada, K. & Kataoka, K. A Facile Amino-Functionalization of Poly(2-Oxazoline)s' Distal End through Sequential Azido End-Capping and Staudinger Reactions. *European Polymer Journal* **88**, 553-561 (2017)
6. Pak, J. K. & Hesse, M. Synthesis of Penta- *N* -Protected Homocaldopentamine and Its Selective Acylation. *The Journal of Organic Chemistry* **63**, 8200-8204 (1998).
